# Acute childhood diarrhoea in northern Ghana: epidemiological, clinical and microbiological characteristics

**DOI:** 10.1186/1471-2334-7-104

**Published:** 2007-09-06

**Authors:** Klaus Reither, Ralf Ignatius, Thomas Weitzel, Andrew Seidu-Korkor, Louis Anyidoho, Eiman Saad, Andrea Djie-Maletz, Peter Ziniel, Felicia Amoo-Sakyi, Francis Danikuu, Stephen Danour, Rowland N Otchwemah, Eckart Schreier, Ulrich Bienzle, Klaus Stark, Frank P Mockenhaupt

**Affiliations:** 1 Institute of Tropical Medicine and International Health, Charité – University Medicine Berlin, Berlin, Germany; 2 Northern Region Malaria Project, NORMAP, Tamale, Ghana; 3 Department of Infectious Diseases and Tropical Medicine, University of Munich, Munich, Germany; 4 Institute of Microbiology and Hygiene, Charité – University Medicine Berlin, Berlin, Germany; 5 School of Medicine and Health Sciences, University for Development Studies, Tamale, Ghana; 6 Regional Health Administration, Ministry of Health, Tamale, Ghana; 7 Robert Koch Institute, Berlin, Germany

## Abstract

**Background:**

Acute diarrhoea is a major cause of childhood morbidity and mortality in sub-Saharan Africa. Its microbiological causes and clinico-epidemiological aspects were examined during the dry season 2005/6 in Tamale, urban northern Ghana.

**Methods:**

Stool specimens of 243 children with acute diarrhoea and of 124 control children were collected. Patients were clinically examined, and malaria and anaemia were assessed. Rota-, astro-, noro- and adenoviruses were identified by (RT-) PCR assays. Intestinal parasites were diagnosed by microscopy, stool antigen assays and PCR, and bacteria by culturing methods.

**Results:**

Watery stools, fever, weakness, and sunken eyes were the most common symptoms in patients (mean age, 10 months). Malaria occurred in 15% and anaemia in 91%; underweight (22%) and wasting (19%) were frequent. Intestinal micro-organisms were isolated from 77% of patients and 53% of controls (*P *< 0.0001). The most common pathogens in patients were rotavirus (55%), adenovirus (28%) and norovirus (10%); intestinal parasites (5%) and bacteria (5%) were rare. Rotavirus was the only pathogen found significantly more frequently in patients than in controls (odds ratio 7.7; 95%CI, 4.2–14.2), and was associated with young age, fever and watery stools. Patients without an identified cause of diarrhoea more frequently had symptomatic malaria (25%) than those with diagnosed intestinal pathogens (12%, *P *= 0.02).

**Conclusion:**

Rotavirus-infection is the predominant cause of acute childhood diarrhoea in urban northern Ghana. The abundance of putative enteropathogens among controls may indicate prolonged excretion or limited pathogenicity. In this population with a high burden of diarrhoeal and other diseases, sanitation, health education, and rotavirus-vaccination can be expected to have substantial impact on childhood morbidity.

## Background

Diarrhoea is a major cause of childhood morbidity and mortality in socio-economically developing countries. More than one billion episodes of diarrhoea occur every year among children under five years of age causing approximately 2.5 million deaths [1,2] The WHO Child Health Epidemiology Reference Group estimates that 16% of deaths in African children younger than five years are directly attributable to diarrhoeal diseases [3].

Acute diarrhoea in tropical countries can be caused by a broad spectrum of viral, parasitic, and bacterial enteropathogens. For many sub-Saharan countries, studies on the prevalence and clinical significance of different diarrhoeal pathogens are incomplete or not available at all. The contribution of the various pathogens to diarrhoea may differ substantially between regions depending on local meteorological, geographic, and socio-economic conditions. Underlying reasons for the spread of diarrhoeal diseases are found in poor hygiene and sanitation, limited access to safe drinking water as well as in inadequate education of health care providers and recipients [4,5].

In parts of northern Ghana, rotavirus-infection is known to be a main cause of childhood diarrhoea [6-8]. However, its role may differ locally, and the epidemiological, clinical, and microbiological aspects of acute childhood diarrhoea are not well established so far. The aim of the present study was to determine a large spectrum of enteropathogens causing acute childhood diarrhoea in an urban setting in the north of Ghana, to assess its clinical characteristics, and to investigate possible associations between the causative agents and clinical and epidemiological features.

## Methods

### Study area and population

The Northern Region of Ghana has in all parts savannah-type vegetation and climate with rains from May to October. Malaria is hyperendemic with little seasonal variation [9]. Tamale, the regional capital, has a population of approximately 350,000 inhabitants but a rather rural character with hamlets and thatched, mud-wall huts scattered over a vast area.

Between November 2005 and January 2006, i.e., during the dry season, 243 children with acute diarrhoea were consecutively enrolled at the Bulpeila Health Centre. The Bulpeila Health Centre, a study site of the Institute of Tropical Medicine Berlin, is divided into a research section and an outpatient and maternity clinic. The facility is providing primary health care for the approximately 50.000 adults and children of Bulpeila subdistrict, the catchment area of the study. The population structure and the rural character of Bulpeila is representative for the entire Northern Region where the dominant ethnic group are the Dagomba who speak Dagbani and are mainly Muslims. As in the whole region the socio-economic standard of Bulpeila's population is low and subsistence farming and small scale trade are the prevailing sources of income.

Patients met the following inclusion criteria: acute diarrhoea (≥ 3 watery or loose stools within 24 hours for < 14 days [10]), age ≤ 12 years, absence of diseases requiring referral to hospital, no anti-infective therapy 48 hours prior to recruitment, and informed written consent from the children's guardians. The study was designed with a broad target age of 0–12 years because an analysis in different age groups was intended and the actual age distribution of children with acute diarrhoea attending Bulpeila Health Centre was not known in advance. 445 children fulfilled the inclusion criteria andof those 243 (54.6%)wereenrolled into the study, because stool specimen could be obtained at the study site. In addition, 124 control children, in similar age groups as the patients and residing in the same part of Tamale were recruited at four nurseries and during routine Child Welfare Service at Bulpeila Health Centre. Controls were recruited if they had no acute or chronic diarrhoea, according to the WHO definitions [10], did not receive anti-infective therapy in the last 48 hours, and if verbal consent was obtained from their guardian. The age of patients and controls were documented on weighing cards of the Child Welfare Clinic. The study protocol was approved by the Ethics Committee, University for Development Studies, Tamale.

### Study execution

Patients were clinically examined by the study physician (FAS) and a medical history was obtained. Fever was defined as an axillary temperature > 37.4°C, and underweight and wasting by z-scores of < -2 for weight-for-age and weight-for-height, respectively [11]. Epidemiological data were collected using a standardized questionnaire adapted from the Integrated Management of Childhood Illness initiative [12]. Rehydration therapy and specific anti-infective treatment were carried out according to WHO guidelines [10], and all patients received zinc *per os *for 14 days [13,14]. Other diseases were treated according to Ghana Health Service guidelines [15]. Patients were followed up in individual intervals until complete recovery (93.4 %) or loss to follow-up or withdrawal of consent (6.6 %).

### Specimen collection

Trained health workers collected faecal specimens from patients and controls. Specimens were immediately processed. Aliquots were frozen at -20°C and preserved using merthiolate-iodine formaldehyde (MIF) solution. Aliquots were shipped to Berlin, Germany, for further investigation, on dry ice in case of frozen samples. Blood samples from patients were collected into EDTA-coated plastic tubes.

### Laboratory examinations

Full blood counts were performed applying a KX-21N cell counter (Sysmex, Japan). Anaemia was defined as haemoglobin < 11 and < 12 g/dL in children below six and twelve years of age, respectively [16]. Malaria parasites were counted microscopically per 500 white blood cells on Giemsa-stained thick blood films. Submicroscopic infections and *Plasmodium *species were determined by nested PCR assays [17] after DNA extraction (QIAmp, Qiagen, Germany). Rota-, astro-, noro-, and adenoviruses were detected by nested (RT-)PCR assays [18,19]. Intestinal parasites were diagnosed by light microscopical examination of native and MIF-concentrated stool specimens [20]. Furthermore, for the detection of *Giardia lamblia, Cryptosporidium sp*. and *Entamoeba histolytica*, stool antigen assays (MeriFluor Crypto&Giardia, Meridian Bioscience, USA; TechLab *E. histolytica *II, TechLab, USA) were applied. Positive *Cryptosporidium sp*. and *E. histolytica *results were verified by Kinyoun modified acid-fast staining and PCR, respectively [21,22]. Microsporidia were excluded by calcofluor white and Ryan's modified trichrome stains [23,24]. Bacterial pathogens were identified by culturing methods. To detect *Salmonella*, *Shigella*, and *Vibrio *species, standard agar plates and enrichment media were used (MacConkey, XLD, TCBS agars, selenite broth, peptone water). In addition, selective media were inoculated for the detection of *Yersinia*, *Aeromonas*, and *Campylobacter *species (CIN, Ryan, Karmali agars; all Oxoid, Germany). Suspicious bacterial colonies were further isolated and differentiated using routine techniques (e.g., oxidase and motility for *Campylobacter *spp., agglutination for *Salmonella*, *Shigella*, and *Vibrio*) or the API system. All differentiated and all suspicious isolates were stored at -20°C using the cryobank system (Mast Diagnostica, Germany). Additionally, collective samples harvested from MacConkey agar plates were frozen (MacConkey cryobanks). All cryobanks were shipped on dry ice to Berlin, Germany, for further analyses. There, samples from MacConkey cryobanks were spread on routine agar plates for detection and differentiation of enteric pathogens as before. In case of discrepant results, single isolates from cryobanks were further studied.

### Statistical analysis

Data analysis was done using StatView statistical software (SAS Institute Inc., USA). Proportions were compared by χ^2^-tests, and odds ratios (ORs) and 95% confidence intervals (95% CIs) were calculated. Age- and sex-adjusted ORs for diarrhoea comparing cases and controls were calculated by logistic regression models for each potential enteropathogen. A *P *value < 0.05 was considered statistically significant.

## Results

### Epidemiological and clinical characteristics

The characteristics of the 243 patients with acute diarrhoea are shown in Table 1. Despite a target age of 0–12 years, 98 % of patients enrolled were under five(mean age: 0.8 years; range 0–11 years); and both underweight and wasting were seen in one out of five children.

**Table 1:** Epidemiological and clinical characteristics of the children with acute diarrhoea (n = 243)

***Epidemiological and clinical characteristics***		
Age	mean (range) [years]	0.8 (0 to 11)
Female/male	[%]	50.6/49.4
		
**Nutritional status**		
Weight-for-age z-score < -2	[%]	21.8
Weight-for height z-score < -2	[%]	19.4
		
**Number of household members**		
Adults and children	mean (range)	5–7 (2–4 to > 22)
Children under 12 years	mean (range)	1–4 (1–4 to > 16)
		
**Most frequently used water source **		
Public tap	[%]	42.7
Pond, river or stream	[%]	34.0
Protected dug well or protected spring	[%]	10.4
		
**Most frequently used toilet facility**		
Pipe ventilated covered latrine	[%]	46.5
Bush or field	[%]	32.4
Uncovered latrine	[%]	12.4
		
**Symptoms reported by guardian**		
Duration of diarrhoea	mean (range) [hours]	72 (6 to 168)
Stool frequency in the last 24 h	mean (range)	5.5 (3 to 15)
Fever	[%]	83.1
Abdominal pain	[%]	70.4
Vomiting	[%]	66.7
Weakness	[%]	56.0
Cough	[%]	45.7
Restlessness	[%]	25.1
Increased Thirst	[%]	22.6
Other symptoms	[%]	3.7
		
**Drug intake in the last 48 h**		
Paracetamol	[%]	28.4
Chloroquine	[%]	7.8
Other drugs	[%]	2.9
		
**Symptoms and signs at examination**		
Fever	[%]	54.3
Weakness	[%]	28.0
Sunken eyes	[%]	16.0
Restlessness	[%]	7.4
Crepitation (auscultation of lung)	[%]	6.6
Other symptoms and signs	[%]	10.8
		
**Concomitant diseases **		
Malaria	[%]	14.8
Lower respiratory tract infection	[%]	7.0
Skin infection	[%]	5.8
Other concomitant diseases	[%]	5.3
		
**Aspect of stool**		
Watery	[%]	75.3
Loose	[%]	20.2
Semi-formed	[%]	2.5
Formed	[%]	2.1
Mucus	[%]	58.4
		
**Blood tests**		
Malaria parasitaemia	[%]	36.2
White blood cell count	mean ± SD [× 10^3^/μL]	12.0 ± 5.0
Haemoglobin concentration	mean ± SD [g/dL]	9.2 ± 1.8

The household questionnaires revealed that the most frequently used water source was the public tap (43%); one third of the households obtained water from ponds, rivers, or streams. For approximately half of the patients' households, a public pipe ventilated covered latrine was the most frequently used toilet facility whereas one third stated to commonly defecate somewhere out of doors, e.g., in the field (Table 1).

The mean duration of diarrhoeal symptoms as reported by the patients' guardians was 3 days (range, 6 hours to 1 week) and the mean stool frequency in the last 24 hours was 5.5 (range, 3–15). Main reported symptoms were fever, abdominal pain, vomiting, and weakness. On examination, more than half of the patients were found to be febrile, and more than a quarter of patients showed pronounced weakness as a sign of dehydration. Anaemia was present in 90.9%. Moreover, lower respiratory tract infection and skin diseases were common concomitant diseases (Table 1).

Control children had a mean age of 1.5 years (range, 0–10 years). None of these children showed overt signs of clinical disease, and in particular, no signs of acute or chronic diarrhoea as defined before. Still, 65.3% of their stool samples were classified as loose or watery.

### Enteropathogens in patients and controls

Potential enteropathogens were isolated from 186 patients (76.5%) and 66 controls (53.2 %; *P *< 0.0001). A single infectious agent was detected in 50.2% of the patients and multiple infections in 26.3%. Co-infections were significantly more frequent in patients than in controls (26.3%, 64/243 *vs*. 14.5%, 18/124; *P *= 0.01)Enteropathogenic viruses were found in the stool of 178 patients (73.3%) and of 57 controls (46.0%; *P *< 0.0001). Rotavirus was the most common enteropathogen, which was present in more than half of the patients. Adjusting for age and sex, children with acute diarrhoea had eight times increased odds of being infected with rotavirus as compared to control children (*P *< 0.0001; Table 2). In contrast, adenovirus, norovirus, and astrovirus did not differ significantly in frequency between patients and controls (Table 2).

**Table 2:** Potential enteropathogens identified in patients (n = 243) and population controls (n = 124)

	***Study group***	***Control group***	***odds ratio***^b^	***p-value***^c^
***Enteropathogens***	***No. of findings(%)***	***No. of findings (%)***	***adjusted for age and sex (95 % CI)***	
**Viruses**^a^	**178 (73.3)**	**57 (46.0)**	**3.27 (2.1 to 5.2)**	**< 0.0001**
Rotavirus	133 (54.7)	15 (12.1)	7.73 (4.2 to 14.2)	< 0.0001
Adenovirus	67 (27.6)	39 (31.5)	1.12 (0.7 to 1.9)	0.7
Norovirus	23 (9.5)	11 (8.9)	0.84 (0.4 to 1.8)	0.7
Astrovirus	11 (4.5)	2 (1.6)	2.53 (0.6 to 11.7)	0.2
				
**Parasites**^a^	**12 (4.9)**	**16 (12.9)**	**0.56 (0.2 to 1.3)**	**0.2**
*Giardia lamblia*	9 (3.7)	12 (9.7)	0.56 (0.2 to 1.5)	0.2
*Cryptosporidium *sp.	1 (0.4)	1 (0.8)	-	-
*Hymenolepis nana*	2 (0.8)	1 (0.8)	-	-
*Strongyloides stercoralis*	1 (0.4)	1 (0.8)	-	-
*Ancylostoma duodenale*	1 (0.4)	1 (0.8)	-	-
				
**Bacteria**^a^	**12 (4.8)**	**2 (1.6)**	**4.8 (0.9 to 27.0)**	**0.07**
*Salmonella enterica *sp.	6 (2.4)	0 (0.0)	-	-
*Campylobacter *sp.	2 (0.8)	1 (0.8)	-	-
*Shigella sp*.	4 (1.6)	1 (0.8)	-	-

^a ^including double infections

^b ^"-" odds ratio was not computed if n ≤ 1 in study or control group;

^c ^"-" not significant

Intestinal parasites and *G. lamblia *in particular, were observed more than twice as frequently in controls than in patients (Table 2), however, this difference was not statistically significant. *Cryptosporidium *sp., *Hymenolepis nana*, *Strongyloides stercoralis*, and *Ancylostoma duodenale w*ere found only sporadically in both patients and controls.

Bacterial enteropathogens were detected more frequently in patients than in controls, at borderline statistical significance (adjusted OR, 4.8; 95%CI, 0.9–27.0). Among patients, the following bacteria were identified: 2 × *Campylobacter jejuni*, 2 × *Salmonella enterica *Gr.2, 2 × *Salmonella enterica *serovar Typhimurium, *Salmonella enterica serovar *Colindale, Salmonella enterica serovar Typhi, 2 × *Shigella boydii*, 2 × *Shigella flexneri*. In controls, one *Campylobacter coli *and one *Shigella boydii *were isolated. *Yersinia*, *Aeromonas*, or *Vibrio *species were not detected in this study.

### Malaria and enteropathogens

Malaria parasites (exclusively *Plasmodium falciparum*) were found microscopically in 24.8% of the patients (geometric mean parasite density, 6335/μL; range, 31–214,741/μL) and in 36.2% by PCR. Malaria, defined as fever *plus *microscopically confirmed parasitaemia, was seen in 14.8%. *Plasmodium falciparum *infection occurred at similar prevalence in patients without an identified cause of diarrhoea (40.4%, 23/57) and in patients with identified enteropathogens (34.9%, 65/186; *P *= 0.5). However, symptomatic malaria occurred significantly more often in children without enteropathogens (24.6%; 14/57) than in children with isolated enteropathogens (11.8%; 22/186; *P *= 0.018).

### Rota- and adenovirus infection and clinical features

Rotavirus-infection was significantly associated with age: it was seen in 66.1% (84/127) of patients of less than one year of age and in 42.2% (49/116) of older children (*P *= 0.0002). *Vice versa*, adenovirus occurred more often in the older (37.1%, 43/116) than in the younger children (18.9%, 24/127; *P *= 0.002). Finally, rotavirus was the only pathogen, which showed associations with clinical presentation, i.e., infected patients revealed a higher prevalence of fever than uninfected patients (62.4%, 83/133 *vs*. 44.5%, 49/110; *P *= 0.005). This difference was more pronounced when excluding cases of symptomatic malaria (58.7%, 71/121 *vs*. 29.1%, 25/86; *P *< 0.001). In addition, rotavirus infected patients presented more frequently with watery stools (58.5%, 107/183 *vs*. 43.3%, 26/60; *P *= 0.041) than uninfected patients. No associations between parasitic and bacterial pathogens on the one hand and epidemiological and clinical characteristics on the other were observed (data not shown).

## Discussion

Acute diarrhoea is one of the most common diseases and causes of death in young children in sub-Saharan Africa and in other developing areas [3,5,25]. The present study constitutes a comprehensive survey on various enteropathogens in northern Ghana and comprises one of the largest diagnostic approaches using PCR for the detection of enteric viruses in West Africa.

One major finding is the confirmation of rotavirus as the principal cause of paediatric diarrhoea during the dry season in the north of Ghana. Its prevalence among children with acute diarrhoea (55%) is in close accordance with the figure of 53% assessed in the dry season in the near-by Kassena-Nankana district [8]. In contrast, in southern Ghana rotavirus was detected in only 7% of symptomatic preschool children in an all-year survey [26]. The lower prevalence in that survey and other studies [27,28] is most likely due to the diagnostic means applied in those studies, i.e., agglutination tests and enzyme immuno assays. The PCR method used in the present study, however, is much more sensitive and specific than the assays used in the previous studies [29,30].

The present study has several limitations. These include the cross-sectional study design, lack of respective data for the rainy season, and the classification of control children. Although the controls did not meet the criteria for acute or chronic diarrhoea [10] and had not received recent anti-infective treatment they rather reflect a random sample of the paediatric population in the study area than being healthy controls: approximately two thirds of the controls provided watery or loose stools. We decided not to exclude those children from the control group in order to obtain a representative picture of intestinal micro-organisms in the paediatric population and also because of logistical reasons. In fact, potential enteropathogens were detected in more than half of the controls' stool samples, possibly indicating subclinical infections. On the one hand, this high burden of intestinal infections in the paediatric population of the study area corresponds to the poor overall health status as reflected by the proportions of malaria, anaemia, nutritional deficits, and concomitant diseases in the present and preceding studies [9]. On the other hand, the abundance of intestinal micro-organisms hampered the identification of pathogens associated with acute diarrhoea. In any case, the controls of the present study cannot be considered exclusively healthy children, which needs to be taken into account when interpreting our data.

Irrespective of these limitations, children with acute diarrhoea had about eight times higher odds of being infected with rotavirus than children in the control group. Rotavirus infection was particularly frequent in the youngest children. We did not apply a numerical score to grade the severity of episodes as proposed by Ruuska & Vesikari [31] because of absent or presumably unreliable data, e.g. on the number of vomiting episodes. Attempts to apply a modified, limited score produced no meaningful results. Nevertheless, in the present study, rotavirus infection was found to be associated with febrile disease and watery stools. These findings are consistent with previous results from Africa and elsewhere [8,27,32]. For cases of diarrhoea without proof of rotavirus or other pathogens, no associations with clinical or epidemiological features were identified. This is likely due to their comparatively small number. Considering the burden of rotavirus infection in the north of Ghana and elsewhere an early implementation of rotavirus vaccines should have first priority. It is estimated that such vaccines could prevent 5% of all childhood deaths and 40% of all deaths due to diarrhoeal diseases [33].

Enteric adenoviruses, serotypes 40 and 41, have gained acceptance as important causes of childhood gastrointestinal illness [34]. In the present study, adenovirus was common, however, occurred at similar prevalence in patients (28%) and controls (32%). The high proportion of adenovirus among the latter does not necessarily exclude its pathogenic role as faecal elimination may continue for months after adequate humoral immune response [35]. This may also be true for norovirus, which was observed in some 9% among both patients and controls. Recent data indicate that noroviruses contribute to the pathogenesis of childhood diarrhoea [19,36]. Its prevalence in our patients corresponds to recent results from Ghana [36]. Astrovirus was rarely identified suggesting a minor role in childhood diarrhoea in northern Ghana. This accords with findings from Botswana [37] and Malawi [38].

Protozoa and helminths were comparatively rare. The overall low prevalence of *G. lamblia *corresponds to its peak occurrence during rainy seasons [28]. However, *G. lamblia *infected fewer patients (5%) than controls (13%). This could imply limited pathogenicity or asymptomatic carriage. The latter is consistent with studies from Brazil [39] and Nepal [40].

Bacteria were the least common potential enteropathogens. In contrast to southern Ghana [26] and other developing areas [28,41,42], the diarrheogenic bacteria *Salmonella sp., Campylobacter sp. and Shigella sp*. were detected only rarely in patients. An increased number of stool samples (optimally three) per patient and the use of highly sensitive PCR for identification of enteropathogenic *Escherichia coli *in further investigations might give a more specific picture of bacterial gastrointestinal illnesses in the region. Nevertheless, the overall limited role of bacteria in acute diarrhoea in the study area strongly argues against a widespread, uncritical use of antibiotics.

Simultaneous infections with potential enteropathogens occurred in about a quarter of patients. Rotavirus in combination with adenovirus, astrovirus, or norovirus was primarily seen. Co-infections were significantly less prevalent in control children. In an individual case of co-infection, the relative role of a single enteropathogen for the clinical symptoms cannot be determined, i.e., one enteropathogen might only be excreted while another one is causing the acute disease. Concerning enteric viruses it seems that the severity of diarrhoea does not correspond to the number of pathogens [43]. However, the impact of viral and non-viral co-infections warrants further investigation.

Malaria is hyperendemic in the study area [9], and, thus, the prevalence of PCR-proven *P. falciparum *infection of 36% in paediatric diarrhoea patients is rather low. The abundance of home-treatment in this (peri-)urban area is one likely explanation [44]. In a previous study at Bulpeila health centre, 15% of children with uncomplicated falciparum malaria presented with diarrhoea, among other symptoms [45]. In the present study, symptomatic malaria was more frequent among patients without than in patients with identified enteropathogens indicating a potential causative, albeit minor role in acute childhood diarrhoea.

## Conclusion

This study from northern Ghana demonstrates that during the dry season, enteric viruses, and rotavirus in particular, are the leading causes of acute childhood diarrhoea. The children of this region – affected by a high burden of diarrhoea, malnutrition, malaria, anaemia and concomitant diseases – would substantially benefit from community health education, improved sanitation and water supply, and a rapid implementation of a rotavirus vaccine.

## Competing interests

The author(s) declare that they have no competing interests.

## Authors' contributions

KR, RI, KS, UB and FPM designed the study. KR, ESa, PZ and FAS were in charge of recruitment, examination, treatment and follow-up of patients and controls. LA, ADM, ASK, PZ, FD, SD and RNO were responsible for sample collection, storage, transport and laboratory procedures in Tamale. RI, TW, ADM, ESc, FPM did the laboratory analyses in Berlin. KR did the statistical analysis and wrote the manuscript with major contributions from other authors. All authors read and approved the final manuscript.

## Pre-publication history

The pre-publication history for this paper can be accessed here:


